# Twenty-Year Collaboration Between North American and South American Retinoblastoma Programs

**DOI:** 10.1200/JGO.2015.002782

**Published:** 2016-03-30

**Authors:** Guillermo L. Chantada, Ira J. Dunkel, Paula S. Schaiquevich, Edith L. Grynszpancholc, Jasmine Francis, Alejandro Ceciliano, Pedro A. Zubizarreta, Adriana C. Fandiño, David H. Abramson

**Affiliations:** **Guillermo L. Chantada, Pedro A. Zubizarreta**, and **Adriana C. Fandiño**, Hospital Juan P. Garrahan; **Paula S. Schaiquevich**, National Scientific and Technical Research Council; **Edith L. Grynszpancholc**, Fundacion Natali Dafne Flexer, de Ayuda al Niño con Cáncer; **Alejandro Ceciliano**, Clínica y Maternidad Suizo Argentina, Buenos Aires, Argentina; and **Ira J. Dunkel**, **Jasmine Francis** and **David H. Abramson**, Memorial Sloan Kettering Cancer Center, New York, NY

Retinoblastoma is a highly curable neoplasm in the developed world.^1^ In less-developed countries, however, survival figures are lower mostly because of delayed diagnosis and poor treatment compliance.^2^ During the past decades, in middle-income countries, disease-free survival of children with retinoblastoma has improved gradually, but there are many challenges related with advanced disease at diagnosis that introduce specific considerations for management.^3,4^ In that setting, children present with more advanced intraocular disease that frequently needs enucleation and systemic treatment to prevent extraocular dissemination; occasionally, they present with metastatic disease that needs high-dose therapy.^2^ Unlike many other pediatric malignancies, medical evidence needed for the treatment of these children is not available from cooperative groups from higher-income countries (HICs), simply because this condition is virtually nonexistent in HICs and because limited results have been reported by cooperative groups. Thus, evidence is left to be generated in less-developed countries. In addition, eye-conservative treatment is a challenge in this setting, not only because more patients present with more advanced disease but also because of the relatively low availability of sophisticated therapies and training of specialized personnel to use local treatments.^5^

Several models of interactions between HICs and low-income countries and, to a lesser degree, between HICs and middle-income countries were instrumented to bridge the survival gap for children with cancer. Twinning institutions in HICs with centers in low- to middle-income countries have been recognized by the International Society of Pediatric Oncology (SIOP) and other organizations as effective instruments to improve results.^6-9^ In Latin America, the partnership between the St Jude Children’s Research Hospital and other institutions in Europe and Canada and the AHOPCA (Central American Cooperative Group) resulted in a sustainable improvement of local capacities in the Central American countries, which led to improved results.^10,11^ There are many examples of successful collaborative programs for retinoblastoma between HICs and low-income countries.^12,13^ However, there is more limited information about partnerships between HIC and upper–middle-income countries—even less so in the field of retinoblastoma, for which the priorities may be different.^8^

In 1995, after an observership visit (by G.L.C.), a collaboration between a retinoblastoma program at a tertiary care, public pediatric hospital in Argentina (an upper–middle-income country) and a major specialized program in a world-leading referral center for this tumor in the United States was established. Retrospectively, three phases were identified in this cooperation (Table 1), which were based upon decisions taken at strategic moments.

**Table 1:**
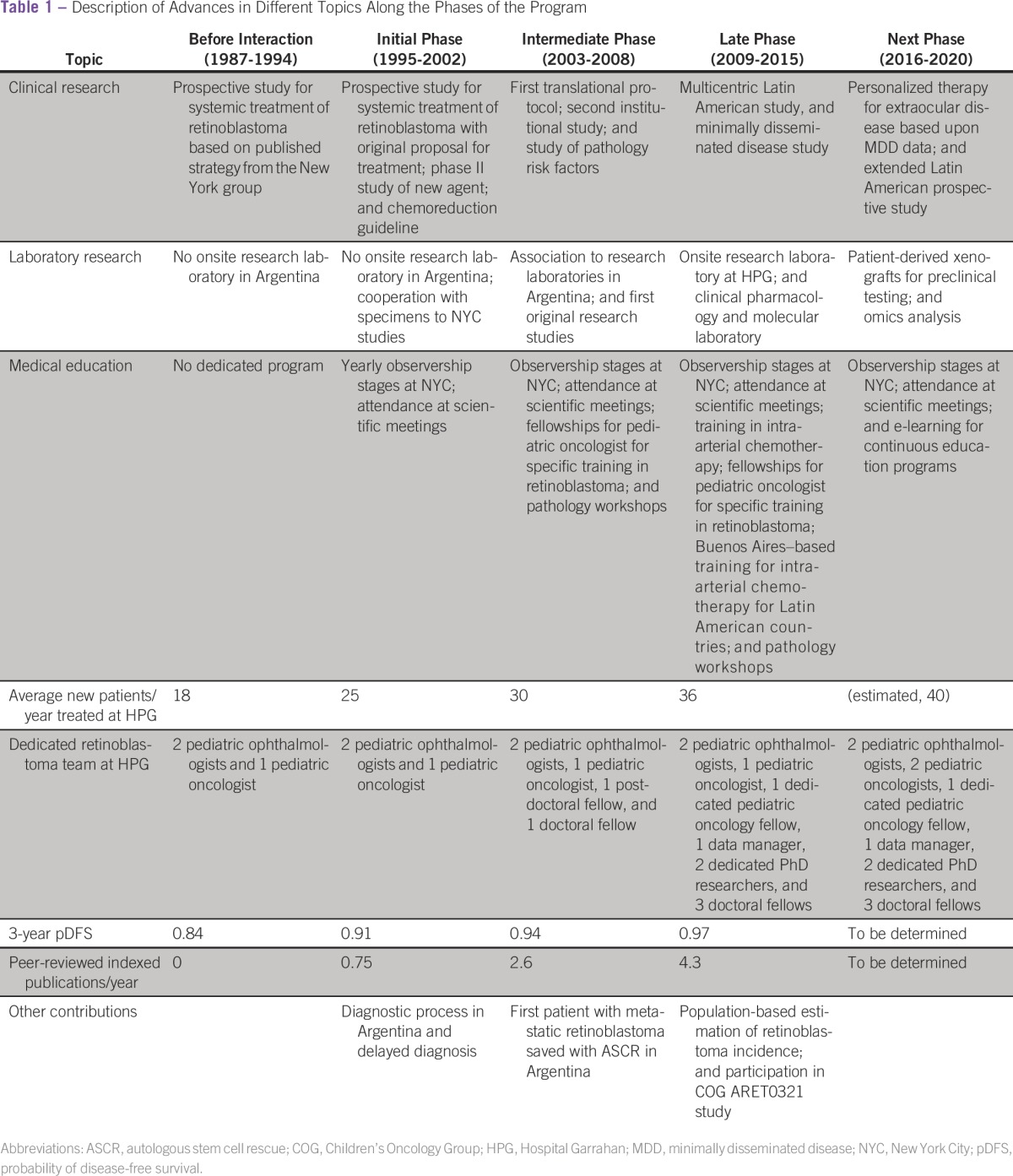
Description of Advances in Different Topics Along the Phases of the Program

The initial phase included the time from 1995 to 2002, during which most actions were directed at medical education through yearly visits (of G.L.C.) to New York and locally implemented baseline actions in Argentina. In this phase, after a review of data and discussions, it was realized that the disease characteristics and local resources were so different in each center that a different strategy should be pursued to improve results in Argentina. Hence, the hallmark for this initial phase was the realization that such differences would require a different approach. The first step, after the results of the first protocol were published,^14^ was the creation of a comprehensive informational patient database for patient registry in Argentina to improve data capture. This provided greater detail about the characteristics of the population under treatment and allowed participants to devise a strategy for improvement of quality of treatment protocol development. During this phase, a phase II study was conducted to identify new drugs, and a treatment protocol was designed on the basis of these learnings.^15^ During this initial phase, most funds from New York City (NYC) were used for financing educational observership visits and attendance at meetings by the Argentine partners. During these observership visits, local Argentine data were discussed with the NYC partners and, on the basis of presented results, future strategic steps were taken. During this time, even though the main objective was to improve patient survival, new treatments, such as chemoreduction aimed to increase eye preservation and diminish late effects, were made available and gradually incorporated in Argentina.^16^ Equipment, such as a fundus camera, was donated by the NYC partner to improve the ophthalmologic evaluation needed for that therapy and to allow image sharing and remote discussion.

The second phase began with the decision to start a translational research program. This phase was also followed by a 3-month stay (by G.L.C.) in NYC. At that time, the NYC group had centralized the patient care at the Memorial Sloan Kettering Cancer Center campus, and a charity, Fund for Ophthalmic Knowledge, was created. From this phase onward, yearly grants were sent to Argentina to support these initial laboratory studies. This also allowed local administration of funds, which made it possible to extend the help to other professionals involved in the care of patients with retinoblastoma in Argentina, and these professionals also were given support for attendance at meetings and observership visits to NYC. This help was administered through an agreement with a nongovernmental foundation in Argentina whose mission is to support the care of families of children with cancer (Fundación Natali Dafne Flexer).

We identify a third phase, which began in 2009, during which these translational research programs matured with the creation of a specific laboratory and the development of higher-impact research, which resulted in publications of laboratory studies and in additional funding to support this research. During this period, the HPG made an additional step and coordinated a prospective study for the treatment of retinoblastoma in Latin America after standards of data capture and management and pathology review comparable to developed countries were achieved. In this phase, intra-arterial chemotherapy was incorporated in Argentina, and the study center was the first center in Latin America to perform this treatment.^17^ To begin this program, a neurosurgeon and an ophthalmologist were trained in NYC before they administered the treatment to patients. With the participation of basic researchers, a more refined production of research studies was undertaken. This included the first animal model for intra-arterial chemotherapy, which enabled estimation of pharmacokinetic parameters for intra-arterial chemotherapy,^18^ and molecular studies, which resulted in the identification of new biomarkers that improved recognition of molecular patterns of disease dissemination.^19^

In this period, translational studies comprised of NYC and Buenos Aires studies were published,^20^ especially in the area of intravitreal and intra-arterial chemotherapy.^21^ All of these developments led to the creation of an ocular pharmacokinetics laboratory in Buenos Aires.

During this period, the Hospital Garrahan became a training hub for Latin American and specialists in other Latin American countries, who came to Argentina, usually with partial support from the Fund for Ophthalmic Knowledge, for training. This resulted, for example, in the implementation of intra-arterial chemotherapy in Brazil, Mexico, Chile, Peru, and Colombia. Global outreach initiatives were led from Argentina with the critical support from NYC, which resulted in the publication of the SIOP guidelines for graduated-intensity treatment.^5^

From 2015 onward, after 20 years of successful interaction, new challenges, such as the implementation of genomic data, newer chemotherapy (and other drugs), ocular pharmacology tested in relevant preclinical models, tumor immunology, and global outreach studies, are envisioned. Human factors are difficult to categorize, but they were critical for the development of this successful program. A fruitful mentorship relationship was cultivated during all of these years by D. H. Abramson, who even supported and directed the PhD thesis of G. L. Chantada, which was defended in 2013 at the University of Buenos Aires.

The involvement of the recipient institution for achievement of long-term sustainability of cooperative programs is important. In that sense, characteristics of the Argentine cancer program were important for achievement of some of the clinical landmarks of this collaboration. For example, oncologic treatment is provided at no cost for all patients at the Hospital Garrahan, and chemotherapy provision is guaranteed by the Argentine governmental agencies or health insurance companies, so there were few limitations for implementation of treatments. During the past decade, the governmental support to medical research in Argentina, especially translational research, increased substantially; that also was a key factor for the success of the program. In the third phase, international initiatives were undertaken from Argentina. Hence, training activities, especially training for in intra-arterial chemotherapy and oncological management, were supported by the fund. The Argentine group coordinated the Latin American protocol for treatment of unilateral retinoblastoma and the participation in the protocol for the Children’s Oncology Group COG 0321 study through GALOP (Grupo de America Latina de Oncologia Pediatrica) in association with the Children’s Oncology Group. In this respect, the group also received support from St Jude Children’s Research Hospital by providing a virtual platform^21a^ for internet meetings for the group. Support from the Cure2children Foundation (Italy) was obtained for data management of that protocol through a connection with the NYC group. For that particular protocol, training sessions for pathologists in each participating institutions to comply with the international definitions^22^ and a dedicated data capture web resource were also supported by the Fund for Ophthalmic Knowledge.

The fund also supported the coordination of the SIOP-PODC (Pediatric Oncology in Developing Countries) graduated-intensity guidelines by providing travel support. The group organized several educational meetings and workshops for information exchange also with support from the Fund of Ophthalmic Knowledge; delegates from Latin American countries attended these meetings and workshops.

In addition, the participation of the Natalie Flexer Foundation as recipient of the grant was not limited to the administration of funds. A booklet for parent information used in NYC was translated to Spanish, and reprints were donated for distribution in Argentina. That was the basis for the creation of a retinoblastoma parental group at the Flexer Foundation, which met regularly on a monthly basis. As a later development, on the basis of the information from the booklet, the group drafted its own booklet and added some local information to provide additional help.

Even though the improvements in survival seen in these 20 years cannot be directly attributed only to this program, a significant improvement in patient survival^23^ and eye preservation was seen in Argentina.^24^ Since the beginning of the interaction, 60 peer reviewed papers (available in PubMed) were published by the Buenos Aires group, and each and every one of them had support from the NYC group. The number of publications per year increased in each period (Table 1). Eighteen of them were collaborative studies between both parts of the interaction.

The program was aimed to build local capacity; as such, fewer than five patients were referred from Argentina to NYC during the period of this interaction, and the rest were treated locally, with few exceptions. From the laboratory research perspective, only one of initial studies included patient specimens sent from Buenos Aires to NYC,^20^ and all of the remaining studies were performed locally in Buenos Aires.

Another indirect indicator of local capacity is the fact that, at the beginning of the program, 100% of the budget was obtained from NYC; currently, approximately 30% of the budget comes from NYC, and the remaining 70% is obtained by the group from competitive local grants. In addition, the overall yearly budget of the group increased six-fold from the beginning of the support for research, whereas the support from the Fund for Ophthalmic Knowledge tripled.

Over time, the degree of development and expertise of the retinoblastoma team in Buenos Aires concentrated the management of the vast majority of the patients with retinoblastoma in Argentina (Table 1). That created a challenge for the Hospital Garrahan, where dedicated staff positions are limited. Even though the retinoblastoma program attracted a number of basic researchers to translational research, the interest of pediatric oncologists and ophthalmologists was more limited, and the program is currently experiencing a shortage of staff. Retinoblastoma has become the most common solid tumor treated at the Hospital Garrahan, and the advent of sophisticated conservative therapies has required more participation of the ophthalmology service and more hours at the operating theaters, which are difficult to obtain in a public pediatric hospital that serves a country-wide population of children up to 16 years.

## References

[1] Broaddus E, Topham A, Singh AD (2009). Survival with retinoblastoma in the USA: 1975-2004. Br J Ophthalmol.

[2] Canturk S, Qaddoumi I, Khetan V (2010). Survival of retinoblastoma in less-developed countries impact of socioeconomic and health-related indicators. Br J Ophthalmol.

[3] Moreno F, Sinaki B, Fandiño A (2014). A population-based study of retinoblastoma incidence and survival in Argentine children. Pediatr Blood Cancer.

[4] Leal-Leal C, Flores-Rojo M, Medina-Sansón A (2004). A multicentre report from the Mexican Retinoblastoma Group. Br J Ophthalmol.

[5] Chantada G, Luna-Fineman S, Sitorus RS (2013). SIOP-PODC recommendations for graduated-intensity treatment of retinoblastoma in developing countries. Pediatr Blood Cancer.

[6] Stefan DC, Shalongo S, Ribeiro R (2012). Twinning in pediatric oncology: An African experience. S Afr Med J.

[7] Howard SC, Marinoni M, Castillo L (2007). Improving outcomes for children with cancer in low-income countries in Latin America: A report on the recent meetings of the Monza International School of Pediatric Hematology/Oncology (MISPHO) —Part I. Pediatr Blood Cancer.

[8] Rodriguez-Galindo C, Wilson MW, Chantada G (2008). Retinoblastoma: One world, one vision. Pediatrics.

[9] Marchevsky DS (2001). Margarita Statement, Sociedad Latinoamericana de Oncologia Pediátrica (SLAOP): Latin American Society of Pediatric Oncology. Med Pediatr Oncol.

[10] Leander C, Fu LC, Peña A (2007). Impact of an education program on late diagnosis of retinoblastoma in Honduras. Pediatr Blood Cancer.

[11] Barr RD, Antillón Klussmann F, Baez F (2014). Asociación de Hemato-Oncología Pediátrica de Centro América (AHOPCA): A model for sustainable development in pediatric oncology. Pediatr Blood Cancer.

[12] Traore F, Togo B, Sylla F (2013). Retinoblastoma: Inventory in Mali and program to develop early diagnosis, treatments and rehabilitation [in French]. Bull Cancer.

[13] Nyamori JM, Kimani K, Njuguna MW (2014). Retinoblastoma referral pattern in Kenya. Middle East Afr J Ophthalmol.

[14] Schvartzman E, Chantada G, Fandiño A (1996). Results of a stage-based protocol for the treatment of retinoblastoma. J Clin Oncol.

[15] Chantada GL, Fandiño A, Mato G (1999). Phase II window of idarubicin in children with extraocular retinoblastoma. J Clin Oncol.

[16] Chantada GL, Fandiño AC, Raslawski EC (2005). Experience with chemoreduction and focal therapy for intraocular retinoblastoma in a developing country. Pediatr Blood Cancer.

[17] Grigorovski N, Lucena E, Mattosinho C (2014). Use of intra-arterial chemotherapy for retinoblastoma: Results of a survey. Int J Ophthalmol.

[18] Schaiquevich P, Buitrago E, Ceciliano A (2012). Pharmacokinetic analysis of topotecan after superselective ophthalmic artery infusion and periocular administration in a porcine model. Retina.

[19] Torbidoni AV, Laurent VE, Sampor C (2015). Association of cone-rod homeobox transcription factor messenger RNA with pediatric metastatic retinoblastoma. JAMA Ophthalmol.

[20] Abramson DH, Frank CM, Chantada GL (1999). Intraocular carboplatin concentrations following intravenous administration for human intraocular retinoblastoma. Ophthalmic Genet.

[21] Francis JH, Schaiquevich P, Buitrago E (2014). Local and systemic toxicity of intravitreal melphalan for vitreous seeding in retinoblastoma: A preclinical and clinical study. Ophthalmology.

[21a] St Jude Children's Research Hospital: Cure4Kids. http://www.cure4kids.org

[22] Sastre X, Chantada GL, Doz F (2009). Proceedings of the consensus meetings from the International Retinoblastoma Staging Working Group on the pathology guidelines for the examination of enucleated eyes and evaluation of prognostic risk factors in retinoblastoma. Arch Pathol Lab Med.

[23] Chantada GL (2011). Retinoblastoma: Lessons and challenges from developing countries—Ellsworth Lecture 2011. Ophthalmic Genet.

[24] Chantada GL, Fandiño AC, Schvartzman E (2014). Impact of chemoreduction for conservative therapy for retinoblastoma in Argentina. Pediatr Blood Cancer.

